# Sense of coherence and intentions to retire early among Finnish women and men

**DOI:** 10.1186/1471-2458-10-22

**Published:** 2010-01-19

**Authors:** Salla-Maarit Volanen, Sakari Suominen, Eero Lahelma, Karoliina Koskenvuo, Markku Koskenvuo, Karri Silventoinen

**Affiliations:** 1 Department of Public Health, University of Helsinki, Finland; 2 Department of Social Policy, Åbo Akademi University, Vasa, Finland; 3 Department of Public Health, University of Turku, Finland; 4 The Local Government Pensions Institution, Medical Affairs and Rehabilitation, Research and Development Unit, Helsinki, Finland; 5 Social Insurance Institution of Finland, Research Department, Helsinki, Finland; 6 Population Research Unit, Department of Sociology, University of Helsinki, Finland; 7 Folkhälsan Research Center, Helsinki, Finland

## Abstract

**Background:**

Previous studies have shown that age, physical and mental health status and working circumstances, along with different socio-economic and psychosocial factors affect the retirement process. However, the role of psychological resources, such as sense of coherence (SOC), on the retirement process is still poorly understood. This study investigated the associations between SOC and intentions to retire early and whether these associations were explained by socio-economic, psychosocial and work and health related factors.

**Methods:**

The data were derived from the Finnish Health and Social Support (HeSSup) Study. The information was gathered from postal surveys in 1998 (baseline) and in 2003 (follow-up). The analyzed data consisted of 7409 women and 4866 men aged 30-54 at baseline. SOC and background factors including childhood circumstances, language, education, working circumstances, social support, health behaviour and somatic and mental health status were assessed at baseline. The intentions to retire early were assessed at follow-up using logistic regression analysis.

**Results:**

SOC was associated with intentions to retire early among both genders. Socio-economic, psychosocial and work and health behaviour related factors did not influence the association between SOC and intentions to retire early among women and men reporting somatic or mental illness. Further, the association between SOC and intentions to retire early remained among (somatically and mentally) healthy men. Among healthy women the association was weaker and statistically non-significant. Among unhealthy women, the odds ratios of SOC was 0.97 (CI 95% 0.96-0.98) and 0.97 among ill men (CI 95% 0.96-0.98), i.e., each additional SOC score reduced the risk of intentions by 3% among both genders.

**Conclusion:**

Unhealthy employees with low SOC and low education were in the greatest risk to have reported intentions to retire early. SOC had an independent effect on intentions to retire early, and a strong SOC may have a potential to prevent early retirement in groups otherwise at risk. An important challenge would be to target the resources of SOC to the most vulnerable and design appropriate interventions in order to strengthen the level of SOC and hence prolong working years of the aging employees.

## Background

The theory of sense of coherence (SOC) was developed in the 1970's as the initiator of the theory, Aaron Antonovsky, examined factors that promote and maintain good health. By focusing on people's resources and capacities to create health, Antonovsky shifted the approach from the pathogenic to the salutogenic. His theory of SOC is the core of that salutogenic model.

SOC is a health promoting psychological resource which strengthens one's capacity to deal with environmental strain [1,2]. Strong SOC is regarded as a characteristic that helps people to identify, use and reuse available resources and therefore minimizes the feelings of tension and stress in life further improving the prospects of staying health. The development of SOC begins in early childhood, and therefore the living circumstances during that time are essential regarding the level of SOC in adulthood. In addition to childhood circumstances, also many other factors called as generalized resistance resources, most importantly education, socio-economic position, social support and work-related factors promote SOC [1,2].

In both cross-sectional and longitudinal study settings, SOC has been found to be positively associated with subjective state of health [3], and negatively with sickness absences [4], circulatory problems [5] and coronary heart disease morbidity and mortality [6-8]. Furthermore, a positive association between SOC and emotional coping and quality of life among people with chronic illness [9,10], health behaviour [11], and self-esteem [12] has been found. In addition earlier research has demonstrated that SOC has a strong negative correlation with anxiety and depression and a strong positive correlation with optimism [13].

### Retirement as a process

The Finnish population is ageing more rapidly than in other OECD countries. At the same time the employment-population ratio for people aged 55-64 is below the EU average and is lower than in Finland's Nordic neighbours: Around 20% of those aged between 55 and 59 are early retired and around 50% of people aged 60-64. The main cause for early exit from the labour market is early disability pension: in 2007 of people aged 60-64 nearly around 24% were receiving disability pension. Additionally, around 14% of pensioners aged 60-64 were receiving unemployment pension. The effective average retirement age in Finland is somewhat below 60 years, although the official retirement age is 63-68 [14].

In the Finnish pension system, the granting of a disability pension requires a medically confirmed illness, disease or injury that essentially restricts or prevents working. Thus medical reasons are evident predictors of disability retirement. However, also other factors, e.g. labour market policy and social protection, affect the relationship between impairment of health and grant of disability pension. Regarding early retirement, an important factor associated with mental and somatic health status is the nature of work since physically and psychosocially demanding and unrewarding working environment is associated with high incidence of early retirement. Therefore alongside age, health status and working conditions are the main reasons for early retirement.

According to Beehr [15], retirement is a process that occurs over a period of time preceding both intention and the decision to retire before the actual retirement age. Both personal factors (e.g. personality attributes, health, skill obsolescence, personal financial states) and environmental factors (e.g. attainment of occupational goals, job characteristics, marital and family situation and leisure pursuits) affect intentions to retire early [15], which further predicts the actual early retirement [16].

Since the intentions to retire early and the actual retirement are different phases of the same process, also the factors associated with them are largely the same. However, also some differences have been found: Especially the characteristics of present work have been highlighted as factors affecting the intentions to retire early [17]. According to a comparative study including 10 European countries, the quality of work and factors presenting well-being (self-perceived health, depressive symptoms, quality of life and number of bodily symptoms) were independently associated with intentions to retire as early as possible [18]. In a Finnish study [19], poor mental health functioning showed a strong and independent association with intentions to retire early. Adjustments for physical health, socio-economic status and spouse's employment status did not essentially affect this association. The same researchers showed in another study [19] that after adjusting for several work and family related psychosocial factors, the association between poor mental health and intentions to retire early halved, yet a clear association remained. Further, the studied psychosocial factors, i.e., high job demands and low job control, low procedural and relational justice, and conflicts between paid work and family life, showed independent associations with intentions to retire early.

### Sense of coherence and intention to retire early

Previous studies, as well as the above mentioned, have shown that several socio-economic and psychosocial factors affect the intentions to retire early, or the associations between health and work related complaints and intentions to retire early, in complicated causal associations [20,17]. With regard to the retirement process, it is noticeable that under the same conditions some people continue working while others begin to consider early retirement. Therefore also people's attitudes and psychological processes and resources have an influence whether a health problem is associated with decreased ability to work or not [21]. Yet, studies on individual differences in terms of personality are sparse. According to a Finnish study [20], the impact of health concerns and aspects of job stress on intentions to retire early varied depending upon occupation and sense of life control: the higher the belief that one can influence one's own life and is responsible for him/herself, and the more favorable the self-image, the less thoughts of early retirement.

To our knowledge only one study has explored the association between SOC and disability retirement, i.e., actual early retirement. A prospective cohort study [21] showed that independently of initial health, a weak SOC of people aged 50 years or less was associated with an increased incidence of disability pension. A few studies have focused on the association between SOC and intentions to retire early. According to Huuhtanen and Tuomi [17] the high level of comprehensibility and manageability components of SOC decreased the intentions to retire early. Rasku [22] and Rasku and Kinnunen [23] showed that weak SOC was associated with intentions to retire early as well as highlighted the push factors as a reason of early intentions. According to Janatuinen [24], strong SOC was associated with the will to continue working until old-age pension. However, these studies addressed only few occupational groups and were based on relatively small (N= 1012-1823) cross-sectional surveys.

### Purpose of the study

In the present study we explore whether SOC is associated with intentions to retire early, and whether socio-economic, psychosocial and work and health behaviour related factors influence this association.

## Methods

### Data

The data were derived from the survey of the 15-year Health and Social Support (HeSSup) study initiated in 1998. The study sample was stratified by gender and age (20-24, 30-34, 40-44 and 50-54 years), comprising three sub samples: Finnish speaking people living in surroundings of the city of Turku, Finland (N = 10 000), other Finnish speaking people in Finland (N = 46 797) and Swedish speaking people in Finland (N = 8 000). The baseline measurements were initiated in 1998 yielding 25 898 valid responses. After one reminder, the response rate was 40%. According to a non-response analysis, late respondents smoked and used psychopharmaceutical drugs more than early respondents, suggesting similar features in the non-respondents [25]. The mail survey was repeated after five years in 2003 with few alterations. It was sent to those who had responded at baseline in 1998, altogether 24 385 individuals. The response rate of the follow-up was 80%. For the purposes of this study, we excluded respondents of the youngest age group (20-24 years at baseline), those who had retired at baseline, those who had strong intentions to retire early at baseline, and those who had already applied for the retirement either at baseline in 1998 or during the follow-up 2003. Thus, the final sample consisted of 12 275 respondents (7409 women, 4866 men, 10882 Finnish speaking, 1393 Swedish speaking Finns).

### Study variables

The outcome variable was intentions to retire early in 2003 and it was asked as follows: 'Have you considered applying for a disability pension, an individual early pension or some other pension?' There were four preset response options: 1) No, I have not considered applying for a pension; 2) Applying for a pension has occurred to my mind; 3) I have strongly considered applying for a pension; 4) I have already applied for a pension. To study the intentions to retire early, those who reported that they had already applied for a pension were excluded. The exclusion of those who have already applied for pension will presumably attenuate the results of the present study since early pension will not be allocated to everyone who apply for it. The dependent variable was thus divided into three categories: no intentions (N = 9778), weak intentions (N = 1824) and strong intentions (N = 673). For further statistical analyses, we combined weak and strong intentions.

SOC and all background variables were measured in 1998. SOC was assessed using Antonovsky's short 13-item scale derived from the original 29 item Orientation to Life Questionnaire (OLQ) covering the three main subcomponents of SOC: comprehensibility, manageability and meaningfulness. The items scored 1 to 7 were randomly ordered in the questionnaire. If missing values existed, they were replaced based on values in other items if at least two-thirds of the questions were answered. The distribution of SOC ranged from 16 to 91. SOC was normally distributed (mean SOC 65.2, standard deviation 11.2). The Cronbach alpha coefficient was 0.85 both in 1998 and 2003 suggesting acceptable internal consistency of the used SOC scale [26].

The quality of the relationship with mother and father in childhood and adolescence was assessed using three categories: 1) Very close and warm or good to both; 2) Very close and warm or good to one, neutral, bad or very bad to the other; and 3) Neutral, bad or very bad to both. Childhood living conditions were measured by six different types of difficulties: Divorce/separation of the parents, long-term financial difficulties in the family, serious conflicts in the family, frequent fear of a family member, alcohol problems, or long-term illness of a family member. All these six questions were assessed as a sum variable with no or at least one difficulty in the childhood home.

Education was classified into four categories: Higher education, higher secondary education, lower secondary education, and primary education only.

Working conditions of the employed were assessed by Karasek's Job Demand-Control Model [27]. Job demands were assessed by a sum variable drawing on five, and skill utilization drawing on six separate questions. Three categories were used: high, average, and low skill utilization [26]. Those currently non-employed, i.e., unemployed, students, home makers and others (n = 3125) were included in the statistical analyses as the fourth category called "others".

Social life included having a partner (yes or no) and perceived social support which was assessed using the Brief Social Support Questionnaire by Sarason et al. [28] with six items. The seven alternative sources of perceived social support were spouse/partner, other close relative, close friend, close friend from work, close neighbour, someone else close to you, no one, and in six different types of situations. The four categories included more than 18 (maximum being 36), 12-17, 6-11, and 0-5 support-givers.

Health-related risk behaviour was measured by alcohol intoxication (drunken once a week or more) and by smoking (non-smoker, former smoker, smoking less than five cigarettes per day and smoking more than five cigarettes per day).

Health status was measured by self-reported somatic disease diagnosed by a physician as follows: Has your physician ever told you that you have or that you have had: Bronchitis, chronic obstructive pulmonary disease, allergic rhinitis, hypertension, diabetes, myocardial infarction, angina pectoris, atricular flimmer/flutter, stroke, other cerebrovascular disorder, peptic/duodenal ulcer, liver disease, renal disease, rheumatoid arthritis, arthrosis, sciatica, glaucoma, epilepsy, cerebral injury, neurological disorder, cancer, some other long-lasting/difficult disease? (Yes/no). Here, two categories included no and yes (has or has had at least one of the diseases).

The Beck Depression Inventory included four classes: No depression (BDI 0-9), minor depression (BDI 10-18), moderate depression (BDI 19-36) and major depression (>36).

For further analyses the data were divided into somatically and/or mentally unhealthy respondents and healthy respondents, i.e., no self-reported somatic disease diagnosed by physician or no reported depression (Beck Depression Inventory).

### Statistical methods

Logistic regression analysis was used to examine the associations of background variables with intentions to retire early. Finnish and Swedish speaking Finns were pooled together since only one statistically significant interaction effect (between language and education with p-value of 0.001) was found. Additionally, weak and strong intentions to retire early were combined. The analyses were made separately among somatically or mentally unhealthy respondents (at least one of the self-reported somatic disease diagnosed by a physician or minor, moderate or major depression), and somatically and mentally healthy respondents (no self-reported somatic disease diagnosed by a physician or no depression). The results are presented as odds ratios (OR) and their 95% confidence intervals (CI). The age-adjusted effects of each variable were first fitted. In addition to age, SOC, language, childhood circumstances, education, working circumstances, social life aspects and health related behaviour factors were adjusted for at the same time. The reference category for the dependent variable, i.e., intentions to retire early, was the first category equalling no intentions. We then divided SOC into quartiles and made the same analysis as in model 2 with background factors adjusted for. Additionally we checked the interactions of SOC with language and gender. Analyses were carried out using the STATA statistical package [29].

## Results

Table 1 shows the prevalences for intentions to retire early by the background variables as well as means of SOC for all intention groups. The mean SOC was strongest among respondents with no intentions to retire early being 65.4 among women and 66.9 among men. Women scored lowest in the weak intention group (mean SOC 63.6) and men in the strong intention group (mean SOC 63.8). Among respondents aged 50-54 years, 42% of women and 53% of men reported some degree intentions to retire early. Finnish speaking women and men reported more often intentions to retire early (weak and strong) compared to Swedish speaking Finns. In both genders, the prevalence of both weak and strong intentions was highest if difficulties in childhood home existed. The prevalence of strong intentions was highest among those women and men who got drunk once a week or more. Among men the effect of smoking on both weak and strong intentions was clear. In both genders having or having had at least one somatic disease diagnosed by a physician and depression strongly increased intentions to retire early. Over 80% of women and men reported no depression, whereas 63% of women and 60% of men reported a previous or present disease diagnosed by physician.

**Table 1:** Intentions to retire early (%) by age, SOC, language, relationship with parents at childhood, difficulties at childhood, education, job demands, skill utilization, having a partner, social support, alcohol intoxication, smoking, somatic disease and depression. Women and men.

	Women			Men			Distribution	
		
	No intentions	Weak intentions	Strong intentions	No intentions	Weak intentions	Strong intentions	Women	Men
**Age**								
30-34	95.6	4.0	0.4	92.8	6.0	1.3	35	32
40-44	90.0	8.7	1.4	85.0	12.4	2.7	36	35
50-54	57.9	28.5	13.5	46.9	36.1	17.0	29	33
							100 (7409)	100 (4866)
**SOC**	**65.4**	**63.6**	**64.4**	**66.9**	**64.8**	**63.8**	**N = 7409**	**N= 4866**
**Language**								
Finnish	82.3	13.0	4.6	74.1	18.8	7.1	81	88
Swedish	86.7	10.0	3.2	80.1	13.6	6.2	9	12
							100 (7409)	100 (4866)
**Relationship with parents at childhood**								
Good to both	83.2	12.7	4.1	76.5	17.2	6.3	60	66
Good to one/neutral or bad to another	83.1	11.9	5.1	71.4	20.5	8.2	28	25
Bad to both	80.3	14.4	5.3	72.8	18.7	8.5	12	9
							100 (7409)	100 (4866)
**Difficulties at childhood**								
No	86.2	10.3	3.5	80.9	14.0	5.1	39	46
Yes (1-6)	82.1	13.2	4.8	72.1	20.7	7.3	61	54
							100 (5666)	100 (3749)
**Education**								
Higher	87.3	10.3	2.3	82.4	14.0	3.6	19	21
Higher secondary	85.9	10.1	4.0	76.2	18.0	5.8	40	28
Lower secondary	81.7	14.8	3.5	76.8	16.5	6.7	19	29
Primary	74.3	17.7	8.1	63.4	24.5	12.1	22	22
**Job demands**								
Low	85.4	11.1	3.4	77.0	16.4	6.6	20	20
Average	83.3	12.6	4.2	76.8	17.2	6.0	41	45
High	80.8	13.9	5.2	72.5	20.7	6.9	20	20
Non-employed							19	15
							100 (7409)	100 (4866)
**Skill utilization**								
High	84.1	12.1	3.9	76.9	17.5	5.6	40	50
Average	82.2	12.8	5.0	73.7	18.8	7.5	28	27
Low	81.8	13.6	4.6	74.0	17.9	8.1	19	15
Non-employed							13	8
							100 (7409)	100 (4866)
**Having a partner**								
Yes	83.3	12.5	4.2	74.3	18.7	7.1	77	79
No	81.1	13.5	5.5	77.4	15.9	6.7	23	21
							100 (7404)	100 (4861)
**Social support**								
18->	87.1	8.9	4.1	82.4	12.8	4.7	11	6
12-17	86.3	10.5	3.1	81.2	13.4	5.5	30	18
6-11	80.5	14.2	5.3	72.9	19.5	7.6	52	63
0-5	78.2	16.7	5.1	72.0	21.5	6.6	7	13
							100 (7373)	100 (4808)
**Alcohol intoxication**								
**Once a week or more**								
No	83.1	12.5	4.4	75.1	18.2	6.7	97	89
Yes	80.3	13.3	6.4	71.2	18.5	10.3	3	11
							100 (7176)	100 (4748)
**Smoking**								
No	82.2	13.6	4.2	79.2	15.3	5.5	51	38
Used to	83.7	11.5	4.8	70.8	20.3	8.9	27	35
< 5/day	83.6	12.8	3.6	80.5	15.9	3.7	3	2
> 5/day	82.3	12.5	5.3	72.8	19.6	7.6	19	25
							100 (6961)	100 (4366)
**Somatic disease**								
No	89.1	8.2	2.6	84.2	12.3	3.3	37	40
Yes (at least one)	79.6	15.1	5.2	69.4	21.5	9.1	63	60
							100 (7261)	100 (4774)
**Depression (BDI-21)**								
No	84.5	11.3	4.0	77.1	16.6	6.2	80.2	84.4
Mild	77.6	16.4	5.8	63.9	25.3	10.7	15.9	12.7
Moderate	65.1	27.8	6.9	62.1	27.2	10.6	3.4	2.7
Severe	76.7	11.6	11.6	50.0	30.0	20.0	0.5 (7374)	0.2(4827)

In the further analyses we combined weak and strong intentions. Since both somatic and mental health problems strongly increased the risk of reporting intention to retire early, further analyses were stratified by health status. Thus analyses were made separately among those somatically or mentally unhealthy women (N = 5882) and men (N = 3784), and healthy women (N = 1527) and men (N = 1082). Of the unhealthy women, 4772 (81%) reported no intention to retire, and 1110 (19%) reported intentions to retire. Among men the corresponding figures were 2715 (72%) and 1069 (28%). Of the somatically and mentally healthy women, 1364 (89%) reported no intentions to retire early, and 163 (10%) reported intentions to retire early. Among men the corresponding figures were 972 (86%) and 155 (14%) (data not shown).

Table 2 presents results of logistic regression models for intentions to retire early (weak and strong intentions combined) among somatically or mentally unhealthy and somatically and mentally healthy women. Among ill women, SOC had a statistically significant association with intention to retire early both in Model 1 age-adjusted and Model 2 with all background factors adjusted for. Regarding model 2 with all the background factors adjusted for, the OR of SOC was 0.97, i.e., each additional SOC score reduced the risk of intentions to retire early by 3% in somatically and/or mentally ill women. Further, socio-economic, psychosocial and work and health behaviour related factors did not influence the association between SOC and intentions to retire early among somatically or mentally unhealthy women; adding the background factors in the model 2 had no influence to the odds ratios of SOC in intentions to retire early.

**Table 2:** Logistic regression models of intentions to retire early (weak and strong intention combined) by age, sense of coherence, language, childhood living conditions, education, working conditions, having a partner, social support, alcohol intoxication and smoking among somatically and mentally unhealthy and healthy respondents.

	Intentions among unhealthy women		Intentions among healthy women	
		
	Model 1	Model 2	Model 1	Model 2
	Age	1+All factors of model 1 are in the analyses at the same time	Age	1+ All factors of model 1are in the analyses at the same time
**SOC**	**0.97 **(0.96-0.97)	**0.97 **(0.96-0.98)	0.98 (0.97-1.00)	0.99 (0.97-1.02)
**Language**				
Finnish	1.00	1.00	1.00	1.00
Swedish	**0.66 **(0.51-0.85)	0.76 (0.55-1.04)	0.86 (0.49-1.51)	1.10 (0.57-2.12)
**Relations with parents**				
Good to both	1.00	1.00	1.00	1.00
Good to one, neutral or bad to the other	1.12 (0.95-1.33)	1.01 (0.81-1.26)	0.84 (0.54-1.29)	0.75 (0.42-1.32)
Bad to both	**1.35 **(1.09-1.67)	1.17 (0.87-1.58)	0.97 (0.51-1.83)	0.61 (0.23-1.60)
**Difficulties at childhood**				
No	1.00	1.00	1.00	1.00
Yes (1-6)	**1.35 **(1.12-1.62)	1.19 (0.97-1.47)	1.24 (0.83-1.86)	1.42 (0.89-2.28)
**Education**				
Higher	1.00	1.00	1.00	1.00
Higher secondary	**1.37 **(1.09-1.72)	**1.42 **(1.07-1.87)	0.90 (0.54-1.49)	0.80 (0.43-1.49)
Lower secondary	**1.77 **(1.38-2.27)	**1.41 **(1.02-1.95)	1.04 (0.58-1.86)	0.98 (0.48-1.99)
Primary	**1.74 **(1.38-2.19)	**1.46 **(1.08-1.99)	1.21 (0.70-2.07)	1.12 (0.54-2.33)
**Job demands**				
Low	1.00	1.00	1.00	1.00
Average	**1.32 **(1.07-1.62)	1.21 (0.94-1.57)	0.97 (0.61-1.53)	1.01 (0.57-1.79)
High	**1.51 **(1.20-1.91)	1.27 (0.95-1.70)	1.24 (0.70-2.18)	1.50 (0.75-3.00)
**Skill utilization**				
High	1.00	1.00	1.00	1.00
Average	1.17 (0.98-1.40)	0.96 (0.76-1.21)	0.86 (0.55-1.34)	0.83 (0.48-1.44)
Low	**1.34 **(1.10-1.64)	0.91 (0.69-1.20)	1.04 (0.63-1.69)	0.94 (0.48-1.84)
**Having a partner**				
Yes	1.00	1.00	1.00	1.00
No	1.08 (0.91-1.28)	0.95 (0.76-1.18)	0.94 (0.61-1.46)	0.91 (0.53-1.58)
**Social support**				
18->	1.00	1.00	1.00	1.00
12-17	1.14 (0.86-1.52)	0.95 (0.68-1.33)	1.38 (0.66-2.85)	1.46 (0.60-3.54)
6-11	**1.37 **(1.06-1.78)	0.95 (0.69-1.31)	1.94 (0.98-3.83)	1.96 (0.85-4.51)
0-5	**1.49 **(1.05-2.11)	0.88 (0.56-1.39)	2.86 (1.04-7.8)	2.15 (0.55-8.44)
**Alcohol intoxication**				
**once a week or more**				
No	1.00	1.00	1.00	1.00
Yes	**1.60 **(1.03-2.50)	1.26 (0.70-2.26)	1.46 (0.38-5.64)	3.44 (0.71-16.47)
**Smoking**				
No	1.00	1.00	1.00	1.00
Earlier	0.93 (0.78-1.12)	0.82 (0.65-1.02)	1.18 (0.77-1.80)	0.81 (0.47-1.37)
< 5/day	1.24 (0.79-1.94)	0.87 (0.48-1.57)	1.33 (0.27-6.34)	1.19 (0.23-6.12)
> 5/day	**1.23 **(1.01-1.51)	0.98 (0.76-1.26)	1.22 (0.72-2.08)	0.86 (0.41-1.77)

Odd ratios (OR) and their 95% confidence intervals compared with the "no intentions" class (OR = 1.00). Women.

P < 0.05

As shown in Table 2, among somatically or mentally unhealthy women, after adjustment for the background variables (model 2), in addition to SOC (OR 0.97, 95% CI 0.96-0.98) higher secondary, lower secondary and primary education (OR 1.42, 95% CI 1.07-1.87; OR 1.41, 95% CI 1.02-1.95; OR 1.46, 95% CI 1.08-1.99, correspondingly) remained associated with intentions to retire early. Among somatically and mentally healthy women the association between SOC and intention to retire early was weaker and non-significant, as were the rest of the variables in model 2.

As for women, also among somatically or mentally unhealthy men SOC was associated with intentions to retire early both in Model 1 (age-adjusted) and Model 2 (with all background factors adjusted for) (Table 3). Regarding model 2 with all the background factors adjusted for, the OR of SOC was 0.97 (95% CI 0.96-0.98) i.e., each additional SOC score reduced the risk of intentions to retire early by 3% among somatically and/or mentally unhealthy men. Further, socio-economic, psychosocial and work and health behaviour related factors had almost no influence on the association between SOC and intentions to retire early (the OR of SOC increased from 0.96 to 0.97). However, unlike among somatically and mentally healthy women, among healthy men the association between SOC and intention to retire early remained in both models 1 and 2.

**Table 3:** Logistic regression models of intentions to retire early (weak and strong intention combined) by age, sense of coherence, language, childhood living conditions, education, working conditions, having a partner, social support, alcohol intoxication and smoking among somatically and mentally unhealthy and healthy respondents.

	Intentions among unhealthy men		Intentions among healthy men	
		
	Model 1	Model 2	Model 1	Model 2
	Age	1+All factors of model 1 are in the analyses at the same time	Age	1+ All factors of model 1are in the analyses at the same time
**SOC**	**0.96 **(0.96-0.97)	**0.97 **(0.96-0.98)	**0.96 **(0.94-0.97)	**0.96 **(0.93-0.98)
**Language**				
Finnish	1.00	1.00	1.00	1.00
Swedish	**0.61 **(0.47-0.79)	**0.67 **(0.48-0.94)	0.64 (0.36-1.13)	0.58 (0.26-1.26)
**Relations with parents**				
Good to both	1.00	1.00	1.00	1.00
Good to one, neutral or bad to other	**1.35 **(1.12-1.63)	1.16 (0.90-1.49)	**1.53 **(1.00-2.34)	1.18 (0.65-2.15)
Bad to both	1.15 (0.87-1.52)	0.84 (0.56-1.24)	1.27 (0.66-2.44)	0.67 (0.22-2.00)
**Difficulties at childhood**				
No	1.00	1.00	1.00	1.00
Yes (1-6)	**1.49 **(1.23-1.80)	**1.26 **(1.01-1.57)	**1.78 **(1.16-2.72)	1.32 (0.79-2.20)
**Education**				
Higher	1.00	1.00	1.00	1.00
Higher secondary	**1.67 **(1.30-2.14)	**1.56 **(1.14-2.13)	1.42 (0.83-2.41)	0.97 (0.50-1.86)
Lower secondary	**2.25 **(1.75-2.90)	**1.82 **(1.31-2.54)	1.25 (0.72-2.17)	0.70 (0.34-1.44)
Primary	**2.39 **(1.86-3.06)	**1.80 **(1.29-2.51)	**1.79 **(1.04-3.06)	0.78 (0.36-1.67)
**Job demands**				
Low	1.00	1.00	1.00	1.00
Average	1.05 (0.84-1.31)	1.07 (0.80-1.42)	1.22 (0.75-1.98)	1.18 (0.62-2.24)
High	**1.47 **(1.14-1.89)	**1.46 **(1.05-2.04)	1.74 (0.96-3.16)	1.75 (0.79-3.84)
**Skill utilization**				
High	1.00	1.00	1.00	1.00
Average	**1.38 **(1.14-1.68)	1.11 (0.86-1.44)	1.19 (0.76-1.85)	1.00 (0.54-1.83)
Low	**1.50 **(1.18-1.91)	1.00 (0.72-1.40)	1.4 (0.82-2.47)	1.14 (0.50-2.59)
**Having a partner**				
Yes	1.00	1.00	1.00	1.00
No	**1.24 **(1.01-1.53)	1.02 (0.77-1.35)	1.13 (0.72-1.78)	0.63 (0.31-1.27)
**Social support**				
18->	1.00	1.00	1.00	1.00
12-17	1.28 (0.84-1.94)	1.10 (0.66-1.85)	1.15 (0.43-3.10)	1.19 (0.33-4.29)
6-11	**1.85 **(1.27-2.67)	1.36 (0.85-2.17)	1.33 (0.53-3.32)	1.24 (0.38-4.03)
0-5	**2.10 **(1.38-3.19)	1.15 (0.66-2.00)	0.99 (0.33-2.91)	0.75 (0.17-3.29)
**Alcohol intoxication once a week or more**				
No	1.00	1.00	1.00	1.00
Yes	**1.48 **(1.15-1.90)	0.96 (0.68-1.36)	1.67 (0.89-3.14)	0.96 (0.36-2.53)
**Smoking**				
No	1.00	1.00	1.00	1.00
Earlier	**1.30 **(1.06-1.58)	1.23 (0.96-1.57)	1.09 (0.68-1.74)	1.34 (0.74-2.42)
< 5/day	1.34 (0.69-2.59)	1.32 (0.63-2.77)	0.41 (0.04-3.41)	2.70 (0)
> 5/day	**1.71 **(1.36-2.14)	**1.33 **(1.00-1.76)	**1.65 **(1.02-2.67)	**1.99 **(1.05-3.77)

Odd ratios (OR) and their 95% confidence intervals compared with the "no intentions" class (OR = 1.00). Men.

P < 0.05

Among somatically or mentally unhealthy men (Table 3) in addition to SOC, language, difficulties at childhood, education, high job demands and heavy smoking remained associated in Model 2 with all the background factors adjusted for. Somatically or mentally unhealthy Swedish speakers reported less intentions to retire early compared to Finnish speakers (OR 0.67, 95% CI 0.48-0.94). Further, among unhealthy men, having had difficulties at childhood increased the risk of intention to retire early compared to the group of no difficulties (OR 1.26, 95% CI 1.01-1.57). Also higher secondary, lower secondary and primary education increased the risk of intentions to retire early among unhealthy men compared to highest education group (OR 1.56, 95% CI 1.14-2.13; OR1.82, 95% CI 1.31-2.54; OR 1.80, 95% CI 1.29-2.51, correspondingly). Finally, high job demands showed an association with intentions to retire early (OR 1.46, 95% CI 1.05-2.04), as well as smoking over five cigarettes per day (OR 1.33, 95% CI 1.00-1.76) in Model 2 with all background variables adjusted for.

Among healthy men, in addition to SOC, relationship with parents, difficulties at childhood, primary education and heavy smoking had statistically significant association with intention to retire early in model 1. In model 2 with all background variables adjusted for in addition to SOC, heavy smoking remained associated

We did the same analyses as in model 2 (with all the variables adjusted for) having the SOC scale divided into quartiles to test the linearity of the association. The reference category for the intentions to retire early was the highest quartile. The OR of intentions increased gradually toward the lowest quartile among somatically or mentally unhealthy women: 1.2 (CI 95% 0.96-1.63), 1.5 (CI 95% 1.13-1.98) and 2.0 (CI 95% 1.49-2.66) and among somatically or mentally unhealthy men: 1.0 (CI 95% 0.76-1.35), 1.3 (CI 95% 0.94-1.72) and 1.9 (CI 95% 1.36-2.60), respectively. Further, the parameters of SOC increased gradually also among somatically and mentally healthy men (OR 1.60, 95% CI 0.87-2.94; OR 1.9, 95% CI 0.99-3.8; OR 2.4, 95% CI 0.96-6.03) suggesting a dose-response relationship between SOC and intention to retire early. Among mentally or somatically unhealthy women the two lowest SOC quartiles clearly represented a risk of intention to retire early whereas among men only the lowest SOC quartile had a similar effect.

There was no interaction neither between language and SOC, nor between gender and SOC when predicting early retirement.

## Discussion

SOC was clearly associated with intentions to retire early among somatically or mentally unhealthy women and men, and this association remained even after adjusting for a range of socio-economic, psychosocial and work and health behaviour related background factors. Further, among healthy men, in addition to smoking SOC was the only significant predictor of intention to retire early. Among healthy women no significant association between SOC and intention to retire early was found.

Among unhealthy women, in addition to SOC, age and education were associated with intentions to retire early when all the background factors were adjusted for, i.e. the effects of these factors on intentions to retire early were independent. Among unhealthy men, in addition to these, also language, difficulties at childhood, education, high job demands and heavy smoking were independently associated with intentions to retire early. The effects of education and job demands are evident: Important factors associated with health status are working conditions [20], which often are socioeconomically patterned.

However, due to differences in occupational status, occupational requirements and work and family related psychosocial environment and life situations among employees, the same disease and impairment in a medical sense may lead to different degrees of disability [19]. Further, subjective evaluation of one's health, well-being and resources may affect one's attitudes toward retirement [20]. The strong and independent association between SOC and intentions to retire early is noticeable since after adjusting for several factors known to affect the retirement process, the association between SOC and intentions to retire early did remain constant.

Considering the nature of SOC and salutogenesis, it seems plausible that the level of SOC may determine people's decisions regarding the continuation of work. A person with strong SOC stands the life and its challenges in health promoting manner: without "unnecessary" feeling of stress arising from the confidence that adequate resources will be at hand when needed. It is presumable that this confidential attitude - strong SOC - enhances people's trust that they have the resources needed to overcome the risk factors and challenges posed by their work, and that the work they do is meaningful and important. Whereas a person with a poor SOC may stand his/her work as life in general, it is chaotic, unmanageable, and meaningless, and hence be reluctant to continue working (until old-age retirement).

Additionally research has shown that among people with different kind of chronic illnesses, those having strong SOC deal better with the strain arising from illness [30]. Therefore among people with health complaints, SOC may be a factor preventing early retirement. Additionally, SOC is associated with perceived health [3], self-esteem [13] and attitudes such as optimism and hostility [14]. Optimism/pessimism and hostility have also been associated with the retirement process [31]. Additionally, earlier studies have shown that early rehabilitation is associated with higher average retirement age [32,33]. According to Kaiser et al. [33], SOC is related to coping style and thus is a prerequisite for engaging in rehabilitation.

It has also been shown that the climate at work and working circumstances of people who still are working are highlighted as factors affecting intentions to retire early. In a Finnish study [34] low job control, poor teamwork and unjust supervision were associated with retirement thoughts and retirement preferences among hospital physicians. The association remained after control for indicators of health and social circumstances. In another Finnish study [35] the association between low job control and intentions to retire early was stronger if job demand were high. The present study partly confirmed the role of psychosocial working conditions on retirement process: high job demands at work remained associated with intentions to retire early after full adjustment for other confounding factors among men. According to our earlier research, SOC has been associated positively with ability to use skills, decision authority and skill utilization at work, and negatively with high psychological job demands [26,36]. Moreover, the main effect of SOC on well-being at work and some support for a moderating role of SOC on the relationship between perceived work characteristics and well-being has been reported [37]. To sum up, SOC may influence the retirement process in several ways, i.e. directly determining the process of consideration regarding the continuation of work, or indirectly through better adaptability to ill-health, working circumstances, and engagement in rehabilitation.

Another way to discuss the role of SOC in the retirement process is its role in the retirement developmental transition, especially among people with ambiguous attitudes toward retirement. According to Antonovsky, people with a strong SOC are more prone to substitute work activity with other meaningful activities instead of ending up feeling worthless. Antonovsky et al. [38] showed in their study that a strong SOC is an important factor in coping with the retirement developmental transition for it helps adopting functional attitudes toward the retirement. Further, Smith [39] examined the role of family worldview (incl. SOC) on retirement process and showed that the way family views the world is important for a positive level of individual adaptation as well as family unit adaptation during retirement. It seems that SOC is an important factor not only preventing early retirement process but also promoting the readjustment to the old-age pension.

Since earlier studies have shown that Swedish speaking Finns retire later than their Finnish speaking counterparts [40], the language was included in the present study. Compared to Swedish speaking men, Finnish speaking men reported more often intentions to retire early. This association remained even after full adjustment for the background variables. Hence it seems that Finnish and Swedish speaking men show to some extent different attitudes already in the first phase of the retirement process.

The weakness of the present study is that the response rate of the baseline survey was only 40%. However, according to the non-response analysis, only minor differences in socio-demographic and health-related issues were found between the respondents and non-respondents [25]. Further studies would also benefit from a wider range of family and work related psychosocial factors as well as social support compared to the present study. An advantage of the present study is a large nationwide population sample with prospective study design. Also the response rate of the follow-up was good being 80%.

## Conclusion

According to the present paper SOC has an independent effect on intentions to retire early and therefore a strong SOC may have a potential to prevent early retirement in groups otherwise at risk. In the present study unhealthy people with low SOC and low education were in greatest risk to have reported intention to retire early. Determining SOC would help to identify individuals at risk for early retirement and focus supportive measures more effectively. Moreover, several recent studies [41-44] have supported Antonovsky's original hypotheses that intentional modification of SOC through an intervention is possible. An important challenge would be to target the resources of SOC, including working circumstances and social support, to the most vulnerable and design appropriate interventions in order to strengthen the level of SOC and hence prolong working years of the aging employees. More research on individual choices based on personality and attitudes is needed in order to better understand the multi-faceted mechanisms between SOC and retirement decisions.

## Competing interests

The authors declare that they have no competing interests.

## Authors' contributions

S-MV participated in the design of the study, performed the statistical analyses and wrote the first draft of the manuscript. SS, EL, KK, MK and KS participated in the design of the study, commented the draft versions of the manuscript and helped to improve it. All authors read and approved the final manuscript.

## Pre-publication history

The pre-publication history for this paper can be accessed here:

http://www.biomedcentral.com/1471-2458/10/22/prepub
